# Genomic Evolution of White Spot Syndrome Virus in Shrimp: Insights from Transposon Dynamics

**DOI:** 10.3390/biology14060653

**Published:** 2025-06-04

**Authors:** Zhouquan Li, Guanghua Huang, Jingyi Zhang, Mingyou Li, Zhizhi Liu, Sihua Peng, Rui Wang, Dong Liu

**Affiliations:** 1 China (Guangxi)-ASEAN Key Laboratory of Comprehensive Exploitation and Utilization of Aquatic Germplasm Resources, Ministry of Agriculture and Rural Affairs, Guangxi Academy of Fishery Sciences, Nanning 530021, China; 2 Shanghai Universities Key Laboratory of Marine Animal Taxonomy and Evolution, Shanghai Ocean University, Shanghai 201306, China; 3 Key Laboratory of Aquaculture Genetic and Breeding and Healthy Aquaculture of Guangxi, Guangxi Academy of Fishery Sciences, Nanning 530021, China; 4 Department of Epidemiology and Biostatistics, College of Public Health, University of Georgia, Athens, GA 30602, USA; sihua.peng@uga.edu

**Keywords:** genome, evolution, recombination, genetic diversity, transposon

## Abstract

White spot syndrome virus (WSSV) has emerged as a significant threat to global shrimp aquaculture, causing economic losses because of its rapid spread and high mortality rates. This study aims to elucidate the genetic and evolutionary dynamics of WSSV through a comprehensive genome analysis. Utilizing 27 complete genome sequences sourced from public databases, this study investigates the genetic variability, potential recombination events, and evolutionary patterns of WSSV. Our results identified multiple genomic deletions, 14 novel single-nucleotide polymorphism sites, and variable number tandem repeats across different strains, underscoring the virus’s genetic diversity. A recombination event between freshwater and marine strains highlights a complex transmission pathway, potentially facilitated by aquaculture practices. A phylogenetic tree constructed using ancestral genes suggests that WSSV originated in Southeast Asia and subsequently globally spread, influenced by both natural and anthropogenic factors. Genomic shrinkage of the virus occurred in time series, while the host’s viral infection induced transposon transposition and insertion into the earlier virus genome to provide a basis for genomic shrinkage. Our research emphasizes the importance of advanced molecular characterization and evolutionary models of the virus in understanding the spread of viral pathogens in aquaculture environments.

## Simple Summary

Our study investigates the genetic and evolutionary dynamics of white spot syndrome virus (WSSV), a major pathogen affecting global shrimp aquaculture. We analyzed 28 complete WSSV genome sequences from public databases to explore genetic variability, recombination events, and evolutionary patterns. We identified multiple genomic deletions, variable number tandem repeats (VNTRs), and novel single-nucleotide polymorphisms (SNPs) that contribute to viral adaptation. A significant recombination event between freshwater and marine strains was detected, highlighting complex transmission pathways. Our phylogenetic analysis suggests that WSSV originated in Southeast Asia and spread globally through both natural and anthropogenic factors based on an outgroup. Notably, transposon insertions served as a basis for viral genomic shrinkage, providing new insights into the divergence of WSSV. Our findings emphasize the importance of advanced molecular characterization and evolutionary models for understanding viral pathogens in aquaculture environments.

## 1. Introduction

White spot disease is an epidemic disease affecting farmed shrimp, caused by the white spot syndrome virus (WSSV). This virus is an enveloped, double-stranded circular DNA virus and the only member of the monotypic family Nimaviridae, causing mass mortality to result in severe economic loss in farming shrimp production. The infected shrimp have a survival time of 3–10 days, with up to 100% mortality in the shrimp industry [1]. WSSV was first reported in 1992 in farmed shrimp in Taiwan and China [1] and rapidly spread to Korea, Japan, Vietnam, Thailand, Malaysia, Indonesia, India, Iran, and other Southeast Asian countries [2,3,4]. WSSV has also been found in Central and South Americas, including Ecuador [5,6]. WSSV has been listed by the World Organization for Animal Health as a disease that must be reported upon detection [7]. Outbreaks of WSSV have also been reported in Iran [8]. The virus has a wide host range in aquatic animals, including marine and brackish water crustaceans, freshwater shrimp, penaeids, crabs, and plankton [9]. Because of its extensive spread around the world, the virus has caused economic losses of USD 150 billion, increasing at a rate of USD 1 billion annually, representing approximately 10% of the global shrimp production [10].

The genome of the virus is about 280–309 kb in size, known to be one of the largest animal virus genomes described to date, and has been predicted to contain 184 open reading frames (ORFs) [11], encoding about 180 proteins, of which more than 50 are structural proteins [5,12]. The alignment of viral genome sequences from Thailand, the Taiwan region, and mainland China has revealed differences that could be categorized as insertion/deletion regions, recombination-prone regions, redundant variable number tandem repeat (VNTR) regions, and single-nucleotide variant (SNV) and transposon regions, although these genome sequences share an overall identity of 99% [9]. Many studies have addressed genomic differences and provided their predicted ORFs. However, ORF annotation has been performed by gene-finding programs without a uniform standard, which leads to different genes in the orientation of the same region; furthermore, little is known about the evolutionary history of WSSV because of the monotypic family Nimaviridae. Recently, several novel nimaviral genomes have been identified from crustaceans, which are closely related to but distinct from WSSV [13]. This provides an opportunity to investigate the evolution of WSSV. In this study, we reannotated the ORFs of the viral genome available in the public databases, using the TH strain as a standard, to detect variable genomic regions and to analyze the recombination and evolutionary patterns of WSSV.

## 2. Methods and Materials

### 2.1. Sequence Data Collection

A total of 27 complete genome sequences of WSSV were obtained from public databases, including NCBI (https://www.ncbi.nlm.nih.gov, accessed on 30 December 2024), EMBL (https://www.embl.org, accessed on 30 December 2024), DDBJ (https://www.ddbj.nig.ac.jp/index-e.html, accessed on 30 December 2024), and CNGB (https://db.cngb.org, accessed on 30 December 2024) (Table 1), and the viral genomes were obtained from tissue samples of crustaceans collected from the Americas (Brazil, Mexico, USA, and Ecuador), Asia (Japan, Taiwan, mainland China, Korea, and India), and Oceania (Australia). For Japanese WSSV, 13 complete genomes were de novo assembled using Illumina and Nanopore sequencing [14].

### 2.2. Reannotated ORFs of WSSV in Public Databases

The genome sequence of the TH strain was used as a reference, and its annotated ORFs served as a standard to reannotate the full-length genome sequences of the other 26 strains. Two methods were applied to identify the ORFs of the 26 strains. First, the sequences of the TH genome were excised into fragments according to the ORF to construct a library of ORF fragments, and the genome sequences of the 26 strains were aligned to the library using BLASTn. The hits with 99% query coverage and 99% identity were considered as corresponding ORFs. Second, to identify an incomplete ORF because of the deletion/insertion of large fragments, a 12 bp fragment at each end of these ORFs in the TH genome, but not in the test genome, was used as a query to search the genome sequences in both forward and reverse orientations for the corresponding fragment with 99% identity using a custom Python script (https://github.com/shiomutennoxingran/wssv accessed on 30 December 2024). The 12 bp aligned fragments and their span region together were identified as an ORF for the test genome. Finally, the outputs of the two approaches were integrated into an ORF-annotated file for these strains.

### 2.3. Analysis of Variable Region

The genome sequence of the TH strain was used as a standard because of the early stage of the virus discovery and relatively complete gene annotation [5], and variation regions were detected using multiple sequence alignments and Snapgene with the MAFFT model [15]. Variable types were sorted manually. The identities of the ORFs were calculated using MegAlign v18.0 (www.dnastar.com/software/lasergene/megalign-pro accessed on 30 December 2024), and the average percentages of SNVs that occurred in ORFs were calculated using pi values and MEGA7 with the bootstrap method and the Jukes–Cantor model with a bootstrap value of 1000 [16]. Mutations that occurred in the amino acid sequence of the ORF, resulting in amino acid substitutions that affected protein functions, were assessed using Polyphen2 scores based on HumDiv and HumVar training datasets [17]. The scores ranged from 0 to 1, with lower values indicating less impact on protein functions.

### 2.4. Recombination and Evolution Analyses

Recombination analysis was performed as described in [18]. First, all the genome sequences were aligned using MUSCLE [19]. The aligned sequences were scanned for recombination events, using Recombination Detection Program 4 (RDP4) [20] and simPlot [21]; finally, the potential recombination events that were suggested by seven analyses (RDP, Bootscan, GENECONV, MaxChi, Chimaera, SiScan, and 3Seq) from both RDP4 and simPlot were confirmed via similarity results shared by at least three of these analyses. For the evolution analysis, sequences of genes in the variable region identified above were concatenated into a single sequence and tested for nucleotide substitution saturation using DAMBE7 [22]. The genes for the *Penaeus monodon* endogenous nimavirus (BFCF01000001), which are closely related to WSSV, were used as an outgroup. Phylogenetic trees based on nucleotide sequences were constructed using the maximum likelihood algorithm, with bootstrap values determined by 1000 replicates in MEGA7 [16], and the model was selected based on the lowest delta values of AIC, BIC, and DT calculated using jModelTest [23].

## 3. Results

### 3.1. Variable Regions

**Figure 1:**
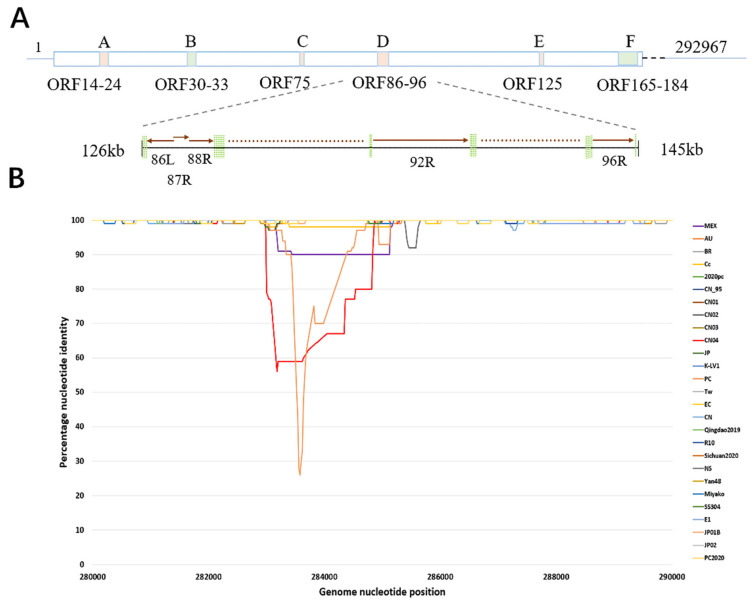
**Schematic representation of the WSSV genome**. (**A**) Patterns of WSSV-TH genomic variant regions. (**B**) Identity map of sequences from variant region F. The upper box indicates the genomic architecture of WSSV-TH, and the variant regions are labelled with letters and corresponding ORFs. Below the solid line shows several genes, and arrows represent the orientations of the gene-encoding protein.

The complete genomes of WSSV revealed six distinct variable regions (Figure 1A). The types of variations mainly included SNV, VNTR, insertion/deletion (InDel < 50 bp), and structural variations (SVs ≥ 50 bp). The variable types were classified into two categories, one comprising variable regions common to all the WSSV strains, including the A (22,865–33,485), C (107,467–108,789), D (126,211–144,687), and E (186,534–188,747) regions, and the other, which consisted of variable regions present in a few strains (fewer than half of the total number of strains), including the B (44,726–51,381) and F (251,400–292,804) regions. The A and C regions showed mainly SVs, and the C, D, and E regions showed variable number tandem repeats (VNTRs), while the B and F regions displayed SNVs and InDels (insertions/deletions). Regions A, B, C, E, and F exhibited sequence identities above 90%, whereas region D showed an 80–89% identity because of the presence of several low-homology genes, including 86L (unannotated), 87R, 88R, 92R (immediate early protein), and 95R (envelope protein) [24] (Figure 1A). The simplot analysis revealed that multiple sequences showed low identities within the F region (Figure 1B).

### 3.2. Variations in ORF Number and Sequences

The total number of ORFs identified in WSSV genomes from different geographical origins ranged from 172 to 184, with 48.3% of the WSSV genomes having 184 ORFs (Table 1). WSSV–Miyako had the fewest ORFs, with 12 missing (56–58, 93–96, 116, 118–120, and 139), followed by the WSSV-E1 genome, with 10 missing (56, 57, 93–96, 118–120, and 145). WSSV-03 and -04 shared the absence of ORFs 161, 162, and 172. ORFs 56–57 were absent in WSSV-07221, -JP03, -JP04, WSSV79, E1, and WSSV14. ORFs 94–95 were absent in WSSV-E1, -AU, -Miyako, -0722-1, WSSV14, -CN03, -CN04, and -Cc. ORFs 161, 162, and 172 were absent in WSSV-CN03 and -CN04. In ORFs 116–121, from one to five ORFs lacked in WSSV-0722-1, -JP04, -E1, -Miyako, WSSV14, -AU, -CN02, -Cc, and -IN. ORFs 129 and 117 were absent in WSSV-Ec and -CN02, respectively. Of these ORFs, 70.4% were 100% identical, 17.6% had an identity ranging from 99% to 90%, and 12% were less than 90% identical, with ORF166 having the lowest identity at 50%.

**Figure 2:**
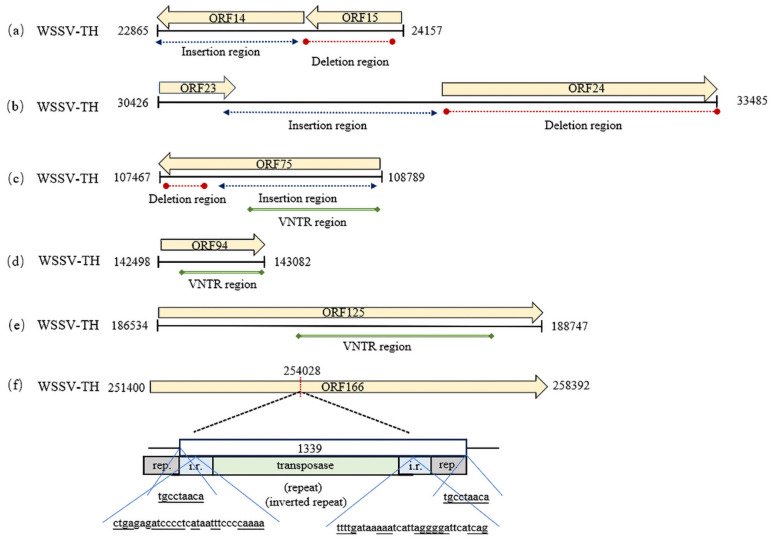
Schematic representation of ORFs in the variant region of WSSV-TH. (**a**) ORF14/15 variation region; (**b**) ORF23/24 variation region; (**c**) ORF75 variation region; (**d**) ORF94 variation region; (**e**) ORF125 variation region; (**f**) ORF166 variation region with transposon insertion.

In ORF14/15, WSSV had insertions in the anterior part of region A, except for the WSSV-Cc, -CN03, -CN04, -AU, and -IN strains (Figure 2), and the size of the insertion fragment ranged from 6 bp (WSSV-BR) to 5368 bp (WSSV- JP01B). An 834 bp insertion in region A was shared by WSSV-CN02, -EC, and -TW, indicating a common ancestral origin. An insertion of 1693 bp occurred in -JP03 and -JP04. Deletions occurred at the middle part of region A in WSSV, except for WSSV- pc2020, -CN01, -BR, -JP01A, -JP02-04, -E1, -0722-1, wssv79, and wssv14. A 757 bp deletion was shared by WSSV-PC, -K-LV1, -CN02, -EC, and -TW. A 635 bp deletion was found in WSSV-Cc, -CN03, -CN04, -AU, and -IN. A 577 bp deletion was observed in WSSV-CN95 and -CN. A 403 bp fragment was lacking in WSSV-MEX.

In ORF23/24, a 326 bp fragment was absent in WSSV-PC, and a 653 bp fragment was deleted in WSSV, except in WSSV-MEX and -PC. Insertions of 2–8 kb sizes occurred in region A of WSSV, except for WSSV-TW, -JP02, and -CN, with insertions of about 13 kb. WSSV-JP03 and -JP04 shared an insertion of 6790 bp (Figure 2).

In ORF75, deletions of 271–751 bp occurred in region C in WSSV-Cc, -CN03, -CN04, -AU, -IN, -K-LV1, -BR, and WSSV-EC (Figure 2). A deletion of 751 bp was shared by WSSV-Cc, -CN03, and -CN04. Insertions of different sizes were identified in region C across WSSV-BR, -EC, -CN02, -pc2020, -TW, -CN95, -CN01, -CN, -JP01A, -JP01B, -JP02, -JP03, -JP04, -E1, -0722-1, -wssv79, and -wssv14. Of these, WSSV-TW showed the largest insertion (519 bp), while WSSV-BR exhibited the smallest (64 bp). A 192 bp insertion was shared by WSSV-EC, -CN95, -CN01, -CN, and -JP01A. Meanwhile, VNTR variations occurred in ORF75, with 1–5 repeat units (RUs) in WSSV, specifically, five identical RUs were observed in WSSV-CN, -CN95, -CN01, -JP01A, -JP01B, and -JP02; another five identical RUs were detected in WSSV-MEX and -PC, while WSSV-IN exhibited two RUs.

A VNTR was observed in ORF94 in region D, except in WSSV-Cc, -0722-1, -E1, -Miyako, -AU, -CN03, -CN04, and WSSV14, which lacked this ORF (Figure 2). WSSV-IN exhibited two RUs, while WSSV-BR contained three RUs. RUs presented in ORF125 in region E of the WSSVs, and most WSSVs had two types of RUs (69 bp and 23 bp), while WSSV-BR had seven RUs classified into three types (69 bp, 69 bp, and 23 bp), and WSSV-IN harbored two RUs belonging to one type (69 bp) (Figure 2). An IS2 transposon of a 1330 bp size was inserted into ORF166 in region F of WSSV-TW (Figure 2).

The lengths of the fragments are indicated as boxes, and insertions/deletions are represented by dotted lines. A VNTR is indicated by a solid line. Rep denotes an inverted repeat, and i.r. indicates a direct repeat. The nucleotide positions with respect to the WSSV-TW genome are symbolized as described in [5].

SNVs were observed in variable regions B and F. Sixteen SNV sites were identified based on a criterion that required single-nucleotide percentages to exceed 30%. Of these, nine SNVs were located in region B (ORF30, 32, and 33), and seven SNVs were located in region F (ORF166 and 167) (Table 2). These single-nucleotide mutations were non-synonymous mutations, including a nonsense mutation in SNV6 and missense mutations at other sites. The values of the allelic frequency of the nucleotide mutation ranged from 0.0526 to 0.9474, and the values of the genotypic frequency were the same as those of the allelic frequency. The functions of the proteins encoded by the mutant sequences were analyzed using Polyphen2, and scores of 0.08–0.30 (Pi > 0.05) indicated that these mutations were neutral mutations unlikely to affect protein functions.

### 3.3. Recombination Analysis

The recombination analysis of the complete WSSV genome sequences was performed using RDP4, and a significant recombination event occurring in the F region was observed between WSSV-PC and WSSV-CN04 (*p* < 0.01), resulting in a recombinant BR that was supported by five analyses of RDP4, including GENECONV (G, P = 3.843 × 10^−16^), MaxChi (M, P = 3.705 × 10^−9^), Chimaera (C, P = 3.735 × 10^−10^), SISCAN (S, P = 4.388 × 10^−23^), and 3Seq (Q, P = 1.388 × 10^−13^) (Table 3). No recombination events were detected using the methods of both RDP (R) and BootScan (B).

**Figure 3:**
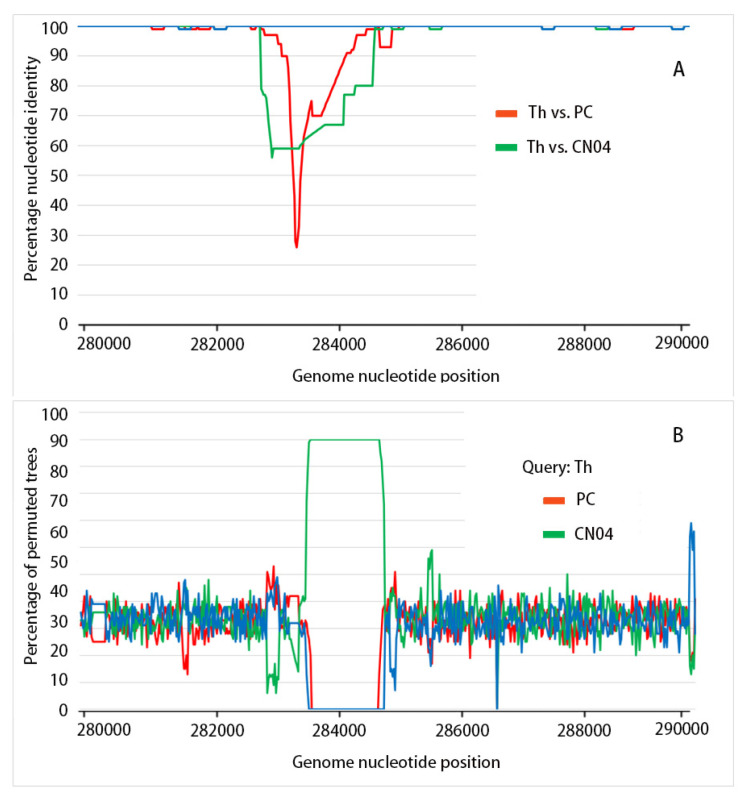
**Detection of recombination events via similarity plot and BootScan analysis**. (**A**) The recombination event was detected using Simplot v 3.5.1 and WSSV-TH as the query sequence. (**B**) The major parent and minor parent were automatically generated in the BootScan software v 3.5.1. All the analyses were performed using a Kimura model, with a window size of 1000 base pairs and a step size of 100 base pairs. The gene map of the query genome sequences was used to accurately position the breakpoints. Blue line indicates the background.

To determine the recombination breakpoints and their start and end positions, WSSV-TH was used as the query sequence, while WSSV-PC and WSSV-CN04 were used as the reference sequences. The results showed that the start and end positions of the breakpoint were 283,413 and 286,292 bp of WSSV-PC, respectively (Figure 3A). This recombination between WSSV-PC and WSSV-CN04 was further confirmed using BootScan analysis (Figure 3B).

### 3.4. Evolutionary Analysis

**Figure 4:**
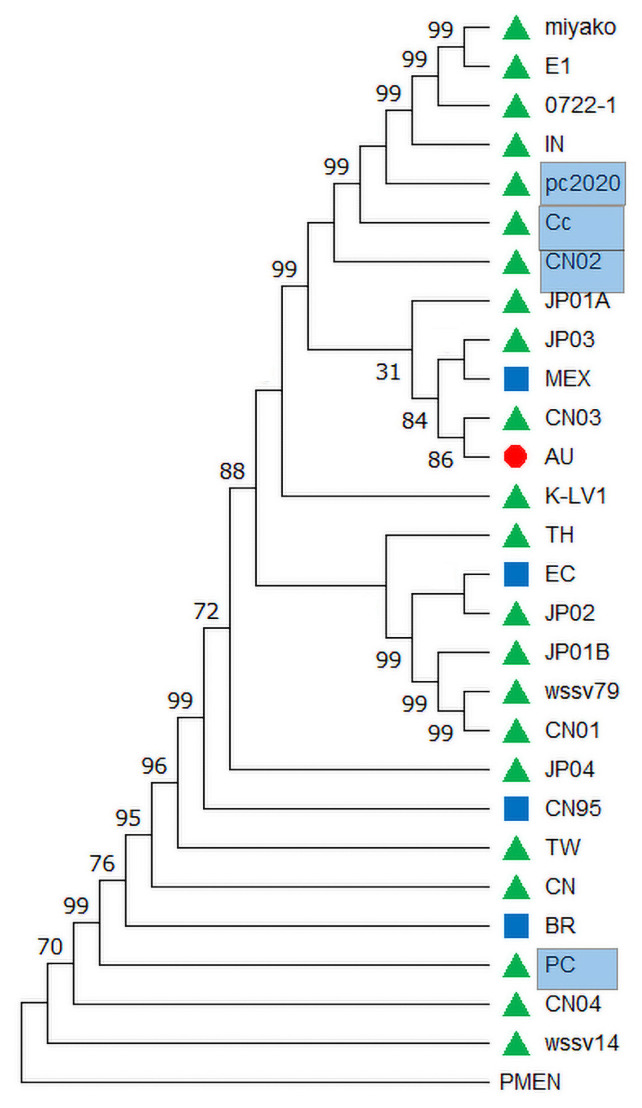
**Evolutionary relationship among WSSVs isolated from shrimp**. The maximum likelihood tree was constructed using MEGA, and the *Penaeus monodon* endogenous nimavirus (PMEN) was used as an outgroup. Strains from freshwater are marked by pale-blue-colored blocks. Strains from the Americas are indicated by blue squares, strains from Oceania by red circles, and strains from Asia by green triangles. The numbers represent confidence levels at the respective nodes.

Because SVs and recombination were lacking in the D variable region, four genes in this region, including 86L, 88R, 92R, and 95R, were used for the evolutionary analysis of the WSSV. The four gene sequences were concatenated, and evolutionary saturation was detected using DAMBE7 [22], with the value ISS = 0.2433 < ISS.c = 0.8355 (*p* < 0.05), indicating that this concatenated sequence was unsaturated for the phylogenetic analysis. The best nucleotide substitution model was evaluated using the jMODEL TEST, and the BIC criterion was selected; finally, the maximum likelihood (ML) method was used to construct the phylogenetic tree using MEGA7. The results showed that the strains isolated from Asia were from an ancient origin and diverged to Australia and America with a high degree of support (99%) (Figure 4). The known freshwater strains, WSSV-Cc, -CN02, and pc2020, clustered in a clade, including WSSV-Miyako, -E1, -0722-1, and -IN, without ORF 87. The recombinant BR evolved from its parent marine strain, WSSV-CN04, and the freshwater strain -PC, as indicated by a high degree of support (99%) at the node of the phylogenetic tree. Recombination between freshwater strains (WSSV-PC) and marine strains (WSSV-CN04) was observed for the first time, providing new clues to the emergence and spread of WSSV.

**Figure 5:**
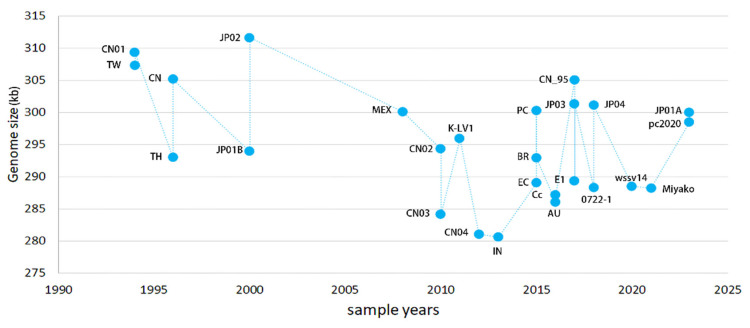
**Dynamics of WSSV genomic shrinkage**. The genome sizes and sample years of the viruses are collected in Table 1.

The strains WSSV-AU and CN03, missing ORF 87, comprised a significant clade in the evolutionary tree (Figure 4). The other strains, WSSV-CN04 and -EC, with the same ORF missing, did not present within a single clade, indicating that ORF-missing events occurred randomly. A clade composed of WSSV-CN01, -wssv79, -JP01B, -JP02, -EC, and -TH was supported by four SNVs, including SNV3, 9, 12, and 14, while the clade composed of WSSV-Miyako, -E1, -0722-1, -IN, -pc2020, -Cc, and -CN02 was supported by two SNVs: SNV13 and 15. In genome sequences of WSSV, pairwise comparison indicated that 51.85% (14/27) of the strains had from one to multiple ORFs missing. Overall, 81.5% (22/27) of the large fragments showed deletions (>400 bp), while 88.9% (24/27) of the strains exhibited insertions. Among the twelve strains isolated earlier (1994–2013), four strains exhibited ORF deletions (33%). Meanwhile, out of fifteen strains isolated later (2015–2023), nine strains exhibited ORF deletions (60%) (Table 1). These deletions indicate that the genome size of the WSSV tends to shrink over time (Figure 5). The genome sizes of the earlier strains exceeded 305 kb, including WSSV-CN01 (309 kb) and -TW (307 kb), with the insertion of transposon IS2 in 1994. Subsequently, the transmission of the WSSV has led to genomic shrinkage, with the smallest being WSSV-CN04 at ~281 kb in 2012.

**Figure 6:**
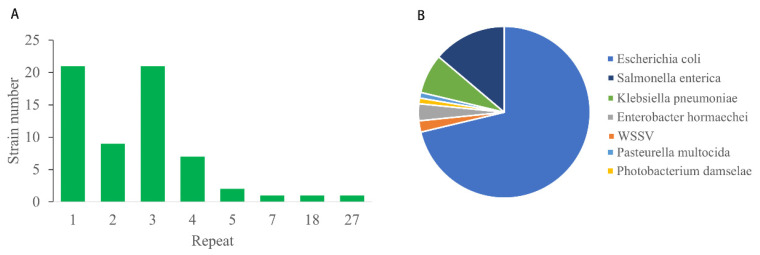
**Distribution of transposon IS2 horizontally transferred between viruses and bacteria**. (**A**) Genomes of *E. coli* strains contain the number of IS2 repeats corresponding to the strain number. (**B**) Composition of species with IS2, as determined in the WSSV genome.

The transposition events observed in the TW strains are rare insertions, and the 100% identity of the IS2 transposase sequences suggests that these were horizontal transposition events. Both ends of transposon IS2 in the TW strains contained a direct repeat sequence (5′-TGCCTAACA-3′). For instance, such direct repeats are located at positions 238,578 and 239,916 in the WSSV-TW genome, suggesting that transposition had occurred. Transposon IS2 exhibits transposition activity. The transposase sequences of IS2 served as a query for a BLAST v 2.15.0 search against the NCBI database, and our discovery revealed that this transposon was widely presented in the genomes of *Escherichia coli*, with at least one copy to a maximum of twenty-seven copies of the repeat in a subset of *E. coli* strains (Figure 6A), indicating that a horizontal transfer occurred between viral and bacterial species. Transposon IS2 has been extensively transferred between different bacterial species at the global scale, with *E. coli* having the highest proportion (67%) and *Photobacterium damselae* and *Pasteurella multocida* having the lowest (1%) (Figure 6B). Thus, transposon IS2, in various bacteria, is possibly capable of integrating into the genomic loci of WSSV strains.

## 4. Discussion

The most significant genotypic changes in WSSV were two genomic deletions in the ORF14/15 and ORF23/24 variable regions, as well as three VNTR loci at ORF75, ORF94, and ORF125 [25]. These variable regions of the WSSV genome have been widely used as molecular markers for WSSV genotyping. Five types of variable regions were detected in farmed and wild shrimp in Madagascar, located in the southwest Indian Ocean, over a 5-year period [26]. In this study, our results confirmed the variable regions, including larger deletions and VNTRs, in ORF75, ORF94, and ORF125. Furthermore, 14 novel SNVs were found in WSSV genomes. Recently, high-frameshift mutations in ORFs have been reported to potentially contribute the severity and spread of WSSV in Japan [27].

Genetic changes in viruses are directly relevant to recombination events [28]. Recombination, as a mechanism for genetic changes, plays an important role in viral evolution and may give rise to new viruses [18]. Using five methods from the RDP4 software v 4.101, a recombination event was detected in the WSSV genome in this study. The recombination region was covered by a region with a low-sequence identity, consistent with previous reports [18]. The recombinant WSSV-BR strain shared an identical 45 bp repeat unit and two SNVs (SNV7 and SNV9) with the WSSV-PC strain but was distinct from the CN04 strain, indicating a recombination event that may have occurred at positions 283,000–284,840 of the WSSV genome. In the recombination event, the parent strains, PC and CN04, were isolated from Asia, and the recombinant BR strain was found in Brazil, indicating that this recombinant strain has undergone long-distance transmission. Interestingly, the PC strain was isolated from freshwater crayfish, whereas the CN04 strain was isolated from marine crayfish, suggesting a link between freshwater and marine crayfish nimaviruses. Previous studies have reported recombination events between other nimavirus strains in freshwater crayfish, such as between the Cc strain and an unknown viral strain at positions 86,200–86,509 and between the CN02 strain and other virus strains at positions 150,750–188,419 in their genomes [24]. These observed recombination events suggest that freshwater crayfish nimaviruses are prone to recombination. The recombination regions in the ORFs contain several envelope proteins (VP190, VP14, VP39A, VP51, VP26, VP16, and VP56) that directly interact with their host counterparts and are essential for nimavirus infection [29].

WSSV is the sole member of the Nimaviridae family, which limits our understanding of its evolutionary history. Recently, an ancestral set of Nimaviridae genes has been identified from other crustacean nimaviruses, and this ancestral gene set shares the phylogenetic origin of the Nimaviridae [13]. These ancestral genes were used as an outgroup, and the phylogenetic tree was constructed using a set of 86L, 87R, 88R, 92R, and 95R genes, revealing the geographic origin and transmission of WSSV in Asia. This pattern is consistent with the hypothesis of a Southeast Asian origin, where the ancestor from Thailand moved to other parts of the world [9]. However, in our phylogenetic tree, the TH strain clusters closely not with the tree’s root but, instead, with the CN03 strain. In our study, the 87R gene is absent in nine strains, and we constructed the evolutionary tree based on four genes, excluding the 87R gene. The TW strain was considered to be the origin of the American WSSV strains, and the first WSSV outbreak was recorded in Taiwan and China in 1994 [1]. This supports the view that the long-distance transportation of shrimp from Taiwan to southern China and other regions played a key role in the spread of WSSV [9]. Interestingly, the phylogenetic tree showed that three strains (Cc, CN02, and pc2020) from freshwater crayfish in Asia were located on a branch, suggesting that they had the same evolutionary pathways. Previous studies have reported that the Pc and CN02 strains were clustered on one branch in a phylogenetic tree constructed using the complete genome sequences of 15 WSSV strains [24]. The phylogenetic tree reflecting epidemic characteristics needs further research.

Deletions frequently occur in viral evolution and may confer benefits for the adaptive evolution of the virus [30]. However, deletions often have a negative impact on viral fitness, reducing viral spread and virulence in the host [31]. In our study, the genome of the TH strain was used as a standard in making pairwise comparisons, and a significant fraction of WSSV strains (77.8%) have large fragment deletions (>400 bp), which have been reported in previous studies [9,26]. The WSSV genome’s size has been reported to shrink over time, and the proportion of host survivors significantly increased as genome size decreased in an aquaculture environment [25]. Genomic shrinkage is not necessarily an adaptive process of viruses, and one of the challenges faced by viruses is to maintain genome integrity during replication and transmission. Recently, it has been reported that the influenza A virus induces the expression of transposons derived from host enhancer regions to regulate the host’s downstream gene expression as a mechanism of virus–host interaction [32]. Several studies have observed the upregulation of transposon expression because of viral infections, such as influenza viruses and numerous other dsDNA viruses [33]. Transposons are discrete genetic elements that have the ability to move and insert themselves into various locations in the host’s genome [34]. The mobile ability of transposons makes them a source of genomic instability; during normal cellular activities, they are repressed by epigenetic chromatin modifications. For a transposon to be mobile, it must first go out of the donor species and integrate into the genome of the new host, using its own gene-encoded transposase, and, therefore, can be transferred extensively between different species without species barriers [35]. It is important to note that transposon insertions into the WSSV genome provided a basis for viral genomic shrinkage. Our results reveal the horizontal transposition of a transposon from *E. coli* into the WSSV genome. This hide-and-seek interplay is central to genomic evolution and speciation.

## 5. Conclusions

In summary, this study has provided insights into the genetic and evolutionary dynamics of WSSV, a major pathogen affecting global shrimp aquaculture. Through detailed genome analysis, we have documented the genetic variability of WSSV and identified SNVs and VNTRs, highlighted by the detection of a recombination event, underscoring the complexity of transmission dynamics. Our findings suggest that the genome size of the WSSV with an Asian origin was shrinking, as deletion mutations during viral spread and transposon insertions provided a basis for genomic shrinkage, thus contributing to the virus’s divergence and persistence in varying hosts and under varying environmental conditions.

## Figures and Tables

**Table 1:** Genomic information of WSSV available in pubic databases.

Accession No.	Abbr.	Length (bp)	ORF Number	Host	Origin	Year
NC_003225	CN01	309,286	184	*Penaeus japonicus*	Mainland China	1994
AF440570	TW	307,287	184	*Penaeus monodon*	Taiwan China	1994
AF369029	TH	292,967	184	*Penaeus monodon*	Thailand	1996
AF332093	CN	305,119	184	*Penaeus japonicus*	Mainland China	1996
AP027279	JP01B	293,923	184	*Penaeus japonicus*	Japan	2000
AP027280	JP02	311,562	184	*Penaeus japonicus*	Japan	2000
KU216744	MEX	300,087	184	*Litopenaeus vannamei*	Mexico	2008
KT995470	CN02	294,261	183	*Procambarus clarkii*	Mainland China	2010
KT995471	CN03	284,148	179	*Litopenaeus vannamei*	Mainland China	2010
JX515788	K-LV1	295,884	184	*Litopenaeus vannamei*	Korea	2011
KY827813	CN04	281,054	179	*Marsupenaeus japonicus*	Mainland China	2012
MG702567	IN	280,591	182	*Penaeus vannamei*	India	2013
MH090824	EC	288,997	183	*Litopenaeus vannamei*	El Guado	2015
KX686117	PC	300,223	184	*Procambarus clarkii*	Mainland China	2015
MF784752	BR	292,912	184	*Litopenaeus vannamei*	Brazil	2015
MF768985	AU	285,973	177	*Penaeus monodon*	Australia	2016
MH663976	Cc	287,179	180	*Procambarus clarkii*	Mainland China	2016
MN840357	CN_95	305,094	184	*Penaeus vannamei*	United States	2017
AP027286	E1	289,353	174	*Penaeus japonicus*	Japan	2017
AP027281	JP03	301,236	182	*Metapenaeopsis lamellate*	Japan	2017
AP027288	0722-1	288,252	176	*Penaeus japonicus*	Japan	2018
AP027282	JP04	301,054	181	*Trachysalambria curvirostris*	Japan	2018
AP027289	wssv14	288,494	175	*Penaeus japonicus*	Japan	2020
AP027290	Miyako	288,190	172	*Penaeus japonicus*	Japan	2021
AP027284	pc2020	298,496	184	*Procambarus clarkii*	Japan	2023
AP027278	JP01A	299,976	184	*Penaeus japonicus*	Japan	2023
AP027283	wssv79	295,104	181	*Penaeus japonicus*	Japan	NA

**Table 2:** SNVs that occurred in the B and F regions in WSSV genomes.

Site	Allelic Frequency	Genotypic Frequency	Type of Mutation	Pi	Score
SNV1 (44276G > A)	G (0.0526)	GG (0.0526)	missense	0.0997	0.142
	A (0.9474)	AA (0.9474)			
SNV2 (46674T > C)	T (0.4737)	TT (0.4737)	missense	0.4986	0.281
	C (0.5263)	CC (0.5263)			
SNV3 (48301G > A)	G (0.2632)	GG (0.2632)	missense	0.3879	0.232
	A (0.7368)	AA (0.7368)			
SNV4 (49066G > A)	G (0.0526)	GG (0.0526)	missense	0.0997	0.102
	A (0.9474)	AA (0.9474)			
SNV5 (49357C > T)	C (0.0526)	CC (0.0526)	missense	0.0997	0.117
	T (0.9474)	TT (0.9474)			
SNV6 (50153A > T)	A (0.8947)	AA (0.8947)	nonsense	0.1884	0.083
	T (0.1053)	TT (0.1053)			
SNV7 (51086A > C)	A (0.0526)	AA (0.0526)	missense	0.0997	0.154
	C (0.9474)	CC (0.9474)			
SNV8 (252108 C > T)	C (0.4737)	CC (0.4737)	missense	0.4986	0.065
	T (0.5263)	TT (0.5263)			
SNV9 (252937 C > T)	C (0.8947)	CC (0.8947)	missense	0.1884	0.095
	T (0.1053)	TT (0.1053)			
SNV10 (257897T > G)	T (0.8947)	TT (0.8947)	missense	0.1884	0.161
	G (0.1053)	GG (0.1053)			
SNV11 (258889A > C)	A (0.4737)	AA (0.4737)	missense	0.4986	0.253
	C (0.5263)	CC (0.5263)			
SNV12 (259660A > G)	A (0.4737)	AA (0.4737)	missense	0.4986	0.153
	G (0.5263)	GG (0.5263)			
SNV13 (265557A > G)	A (0.4737)	AA (0.4737)	missense	0.4986	0.129
	G (0.5263)	GG (0.5263)			
SNV14 (267494G > C)	G (0.0526)	GG (0.0526)	missense	0.0997	0.203
	C (0.9474)	CC (0.9474)			

**Table 3:** Recombination events observed in WSSV.

Recombination Event	Start~End at WSSV-TH	Major Parent	Minor Parent	Analysis (** Denoted *p* < 0.01)						
				R	G	B	M	C	S	Q
WSSV-BR	283,000–284,840	WSSV-PC	WSSV-CN04	-	**	-	**	**	**	**

Note: ** denotes significant recombination observed in the analysis of this model.

## Data Availability

All the data generated or analyzed during this study are included in this published article and at NCBI (www.ncbi.nlm.nih.gov, accessed on 30 December 2024), also seen in Table 1.
